# Analysis of 30-Day Postdischarge Morbidity and Readmission after Radical Gastrectomy for Gastric Carcinoma: A Single-Center Study of 2107 Patients With Prospective Data

**DOI:** 10.1097/MD.0000000000000259

**Published:** 2015-03-20

**Authors:** Oh Jeong, Young Kyu Park, Mi Ran Jung, Seong Yeop Ryu

**Affiliations:** From the Department of Surgery, Chonnam National University Hwasun Hospital, South Korea (OJ, YKP, MRJ, SYR).

## Abstract

PD morbidity and readmission pose a substantial clinical and economic burden to the healthcare system. Comprehensive PD complications and readmission data are essential for developing initiatives to improve patient care. No previous studies have extensively investigated PD complications after gastric cancer surgery.

We investigated the incidence, types, treatment, and risk factors of 30-day postdischarge (PD) complications after gastric cancer surgery.

Between 2010 and 2013, data concerning complications and readmission within 30 days of hospital discharge were prospectively collected in 2107 patients undergoing gastric cancer surgery.

In total, 1642 patients (77.9%) underwent distal gastrectomy, 418 (19.8%) total gastrectomy, and 47 (2.3%) other procedures. Postoperative morbidity and mortality were 17.4% and 0.6%, respectively, with a mean 8.8-day hospital stay. Sixty-one patients (2.9%) developed 30-day PD morbidity (58 local and 3 systemic complications), accounting for 16.6% of overall morbidity; 47 (2.2%) were readmitted; and 7 (0.3%) underwent a reoperation. The mean time to PD complications was 9.5 days after index hospital discharge. The most common complication was intra-abdominal abscess (n = 14), followed by wound, ascites, and anastomosis leakage. No mortality occurred resulting from PD complications. In the univariate and multivariate analyses, underlying comorbidity (hypertension and liver cirrhosis) and obesity were independent risk factors for developing PD complications.

The early PD period is a vulnerable time for surgical patients with substantial risk of complication and readmission. Tailored discharge plans along with appropriate PD patient support are essential for improving the quality of patient care.

## INTRODUCTION

The early postdischarge (PD) period is a vulnerable time for surgical patients with substantial risk of complication and readmission after hospital discharge. In the United States, nearly 15% of surgical patients in Medicare fee-for-service programs were found to be readmitted within a month after the index hospitalization.^1^ Another large study including 21 groups of inpatient general surgical procedures showed that nearly 42% of postoperative complications developed within 30 days after hospital discharge, which more than tripled the risk of reoperation and mortality in surgical patients.^2^ Given its substantial clinical and economic burden to the patient and healthcare system, reducing PD complications and readmissions has become a major target for saving healthcare costs and improving the quality of patient care.^3,4^ Comprehensive and procedure-specific analysis of rates and types of PD complications are essential to develop appropriate interventions to reduce PD morbidity and readmission.

For the past few decades, significant advances have been made in understanding the pathophysiology of postoperative recovery.^5^ More recently, with the introduction of evidence-based surgical care, the length of hospital stay has significantly reduced for major abdominal surgeries. However, as inpatient lengths of stay are decreasing, concerns have been raised over the need for proper care to avoid complications and readmission after hospital discharge. Although most studies reported no significant increase in readmission rates under early discharge programs compared with conventional care,^6,7^ hospital readmissions after early discharge have been reported up to 20% after colonic surgery.^8^ To reduce adverse PD events, hospital discharge should be cautiously decided after accurate patient assessment at the time of hospital discharge. Furthermore, appropriate discharge plans, including patient education, close outpatient monitoring, or provision of home healthcare services, are essential for successful early hospital discharge.^9^

Gastric carcinoma is one of the most common malignancies in East Asian countries.^10^ With an increasing incidence of early gastric cancer in Korea and Japan, surgeons are becoming more concerned about improving the quality of surgical care.^11^ Several studies about morbidity and mortality after gastric cancer surgery report acceptable ranges of morbidity and mortality ranging from 14% to 25% and 0.6% to 1.1%, respectively.^12–14^ However, there are very limited data about how frequently or which types of PD complication occur after gastric cancer surgery. Although one previous study analyzing American College of Surgeons National Quality Improvement Program (ACS-NSQIP) data files has reported 30-day PD morbidity after gastrectomy, major surgical complications such as anastomosis leakage and ileus were not captured in the database.^2^ Some studies sought to investigate readmission after gastrectomy but have not provided comprehensive information about PD complications.^15–17^ In the present study, we prospectively collected data concerning complications and readmission after hospital discharge and investigated the incidence, types, treatment, and risk factors of 30-day PD morbidity after gastric cancer surgery.

## METHODS

### Study Design and Population

Between April 2010 and March 2013, 2202 consecutive patients undergoing gastrectomy for gastric carcinoma were prospectively evaluated with respect to development of complications and readmission after hospital discharge, and 95 of these patients were excluded owing to non-resection surgery (n = 43), preoperative chemotherapy (n = 27), emergency surgery for bleeding or perforation (n = 12), and loss to follow-up (n = 13). The remaining 2107 patients who underwent elective surgery and had complete PD data were included in the analysis. This study was performed with approval of the institutional review board of Chonnam National University Hwasun Hospital, South Korea, which waved informed consent from patients.

### Operation and Postoperative Care

Patients underwent gastric resection and lymph node dissection (LND) according to the Japanese treatment guidelines for gastric carcinoma (2010, version 3).^18^ D1 + LND was performed for early gastric carcinoma and D2 LND for advanced gastric carcinoma. Laparoscopic surgery was indicated for cT1 tumors without LN metastasis. Billroth I, Billroth II, or Roux-en-Y gastrojejunostomy was performed at the surgeon's discretion after distal gastrectomy. Esophagojejunostomy without pouch or interposition was routinely performed after total gastrectomy.

Postoperatively, patients were managed using a standardized clinical pathway protocol. Briefly, preoperative fasting or mechanical bowel cleansing was avoided. Neither a nasogastric tube nor abdominal drain was routinely used. Single-dose prophylactic antibiotics were administered during the operation but not postoperatively. Postoperative pain was managed using epidural anesthesia. Thromboprophylaxis was carried out using an intermittent pneumatic compression device. Patients started an oral diet from the first postoperative day and were administered restricted amounts of intravenous fluid (20–25 ml/kg/day) for 3 days postoperatively.

Hospital discharge was decided if patients meet the following discharge criteria: (1) tolerable pain with no analgesics or oral analgesics only; (2) ability to fully ambulate without assistance; (3) no abnormal physical signs, such as fever, tachycardia, or abdominal tenderness; (4) no abnormal laboratory findings; (5) ability to consume an oral meal without gastrointestinal symptoms; and (6) willingness to go home.

### Postdischarge Survey and Data Collection

Data concerning complications and readmission after hospital discharge were prospectively collected, along with patient's demographic features, operative procedure, pathologic stage, hospital courses, morbidity, and mortality. Overall morbidity and mortality were defined as complications or death during hospitalization or until 30 days after hospital discharge. The 30-day PD morbidity was defined as adverse events occurring between discharge and 30 days after discharge.^19^ The 30-day PD morbidity data were collected with regular follow-up visits 2 weeks and 1 month after hospital discharge. Before hospital discharge, patients were asked to report to our clinic when they experienced any adverse event such as fever, wound discharge, or abdominal pain. Any complications treated in other hospitals were also recorded in the database.

Comorbidities were investigated with respect to hypertension or diabetes mellitus requiring medication; history of chronic obstructive pulmonary disease, pulmonary tuberculosis, or bronchial asthma; liver cirrhosis defined by radiologic or clinical examinations; ischemic heart disease requiring medication or coronary intervention; and history of cerebrovascular accident, renal dysfunction defined as elevated creatinine level or on dialysis, chronic viral hepatitis, and thyroidal dysfunction requiring medication. Pathologic stages were based on the seventh edition of the Union for International Cancer Control (UICC) tumor node metastasis (TNM) classification of malignant tumors.^20^

### Definition of Complications

The type and severity of complications were recorded as defined in our established guidelines.^21^ Complications severity was graded according to the Clavien-Dindo classification.^22^ Briefly, grade I refers to complications not requiring pharmacologic treatment or surgical, endoscopic, or radiologic interventions; grade II refers to those requiring pharmacologic treatments; grade III complications required surgical, endoscopic, or radiologic intervention; grade IV complications were treated in the intensive care unit because of single-organ or multiple-organ failure; and grade V refers to death of a patient. Local complications were investigated with respect to wound complication, intra-abdominal abscess, pancreatic fistula, pancreatitis, anastomotic leakage, anastomotic stricture, gastric stasis, paralytic ileus, intestinal obstruction, stomach necrosis, intra-abdominal bleeding, gastrointestinal bleeding, ascites, and others. Systemic complications were classified as respiratory, cardiovascular, cerebrovascular, renal, and other.

### Statistical Analysis

All data are expressed as mean ± standard deviation or number with percentage. Student *t* test was used to compare continuous variables and *χ*^2^ test or Fisher exact test for categorical variables. The logistic regression model was used for univariate and multivariate analysis of risk factors for PD morbidity. All statistical analyses were carried out using SPSS version 12.0 for Windows (SPSS, Chicago, IL), and *P* < 0.05 were considered statistically significant.

## RESULTS

This study included 1421 men and 686 women (mean age, 61.2 ± 12.0 years; mean body mass index [BMI], 23.6 ± 3.2 kg/m^2^). Patient characteristics, including mean age and BMI, American Society of Anesthesiologists (ASA) physical status, comorbidities, and tumor stages are shown in Table 1. According to the ASA physical status classification, 667 patients (31.7%) were of status 1, 1363 (64.7%) were status 2, and 71 (3.3%) were status 3. More than 1 comorbidity was observed in 1165 patients (55.3%), and hypertension (34.3%) and diabetes mellitus (18.3%) were the most common. The preoperative check-up revealed 539 (25.6%) with preoperative anemia and 34 (1.6%) with hypoalbuminemia. Nearly half (52.0%) of the patients had tumors in the lower third of the stomach. There were 1460 (69.3%) stage I, 258 (12.2%) stage II, 282 (13.4%) stage III, and 107 (5.1%) stage IV patients according to the seventh edition of UICC TNM classification.

**TABLE 1:**
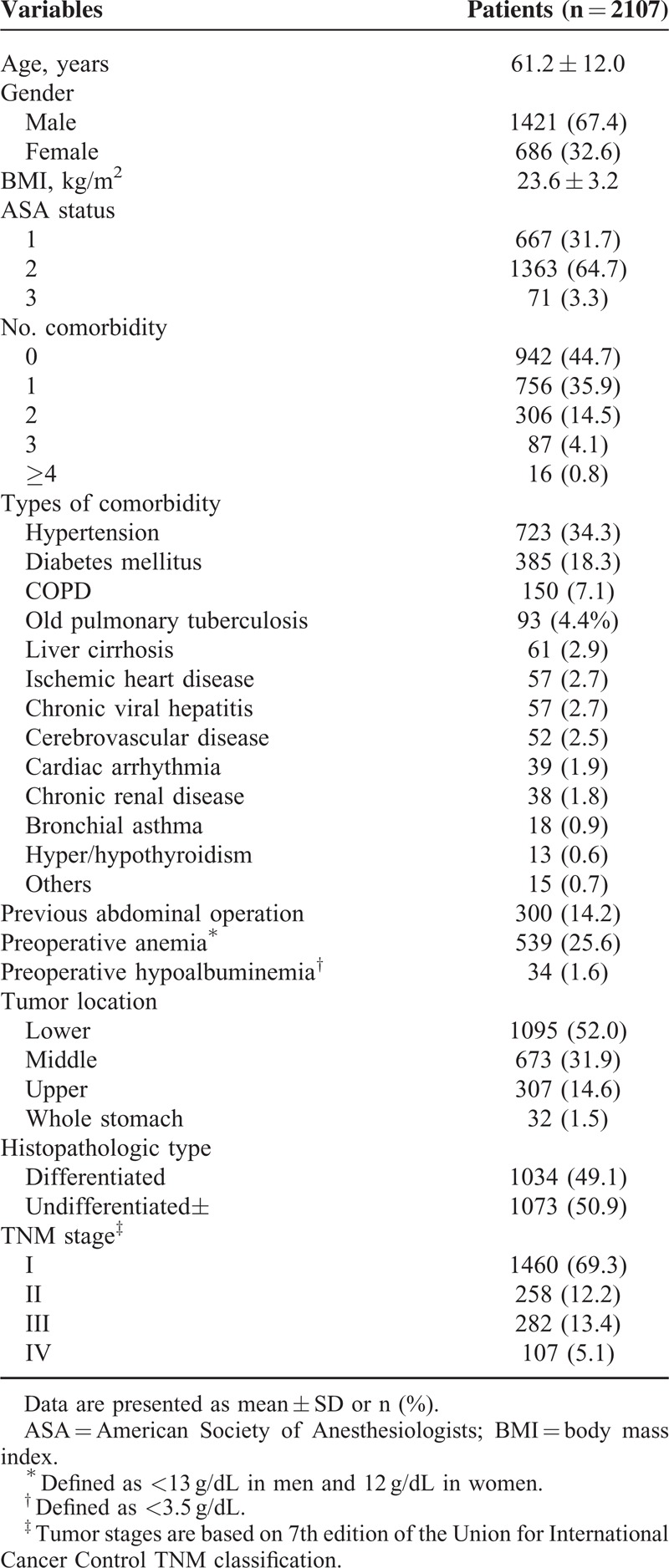
Patient Characteristics

### Operative Outcomes

Operative outcomes are shown in Table 2. Overall, 2043 patients (97.0%) underwent curative (R0) resection; 1642 (77.9%) underwent distal gastrectomy, 418 (19.8%) underwent total gastrectomy, and 47 (2.3%) underwent other procedures. Laparoscopic surgery was performed in 1235 patients (58.6%), D2 LND was performed in 1169 patients (55.5%), and 246 patients (11.7%) underwent combined organ resection, with cholecystectomy (4.7%) being the most common. Most cases of cholecystectomy were performed because of combined gall bladder disease, such as stone or cholecystitis. Other organs like spleen, pancreas, liver, colon, adrenal gland, and ovary were mostly resected for complete oncologic resection. The mean operating time was 195 ± 68 min, and the mean operative blood loss was 180 ± 207 mL. The mean hospital stay was 8.8 ± 7.2 days after surgery.

**TABLE 2:**
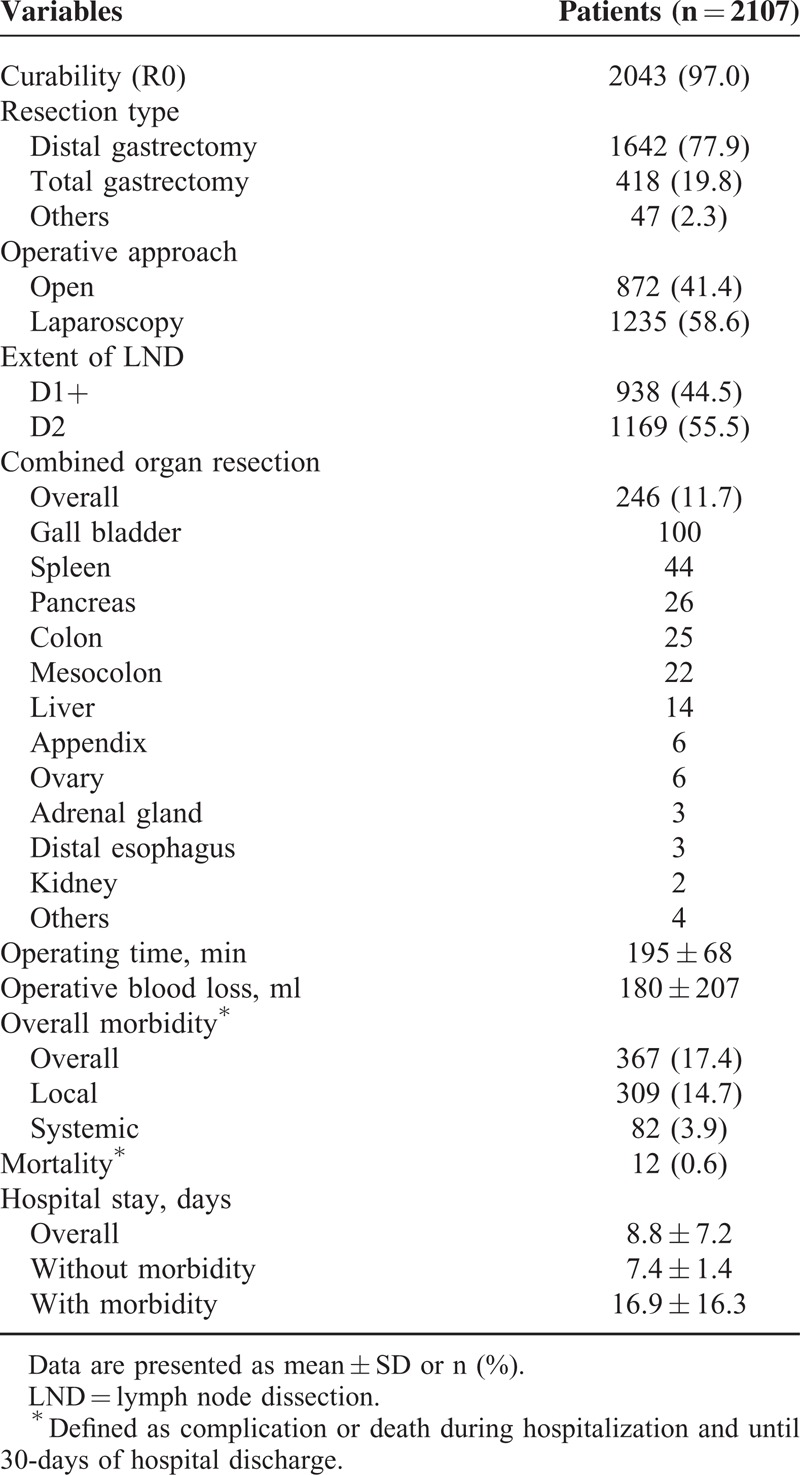
Operative Outcomes

Postoperatively, overall morbidity and mortality were 17.4% (n = 367) and 0.6% (n = 12), respectively (Table 2). There were 309 (14.7%) local and 82 (8.9%) systemic complications in 367 patients. Of the local complications, postoperative ascites (2.3%) were the most common, followed by abdominal infection (2.1%), gastrointestinal bleeding (2.1%), and anastomosis leakage (1.9%). Pulmonary complication (2.5%) was the most common systemic complication.

### Thirty-Day Postdischarge Morbidity

**FIGURE 1:**
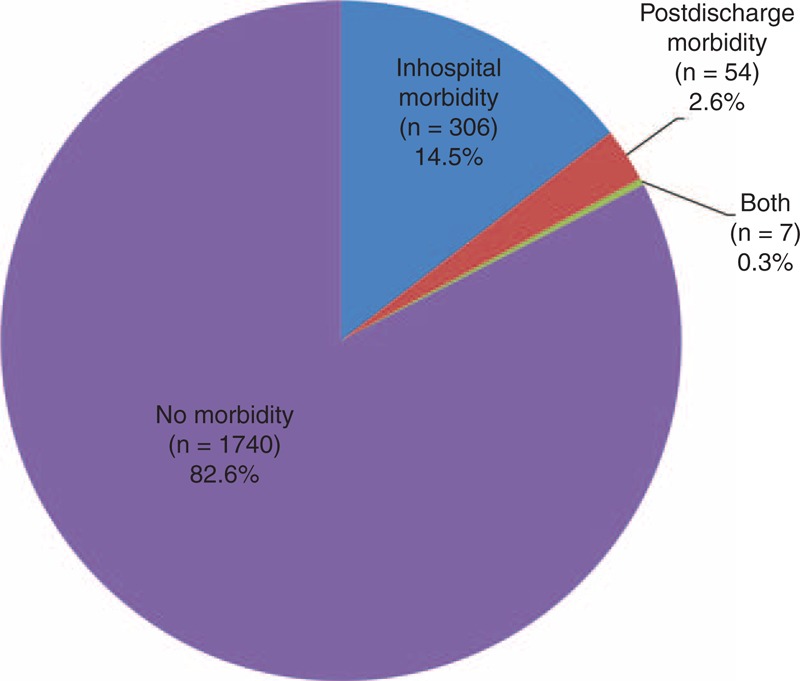
Proportion of PD morbidity. Thirty-day PD complications occurred in 61 patients (2.9%), which accounted for 16.6% of overall morbidity.

Figure 1 shows the proportion of patients with 30-day PD complications. Thirty-day PD complications occurred in 61 patients (2.9%), which accounted for 16.6% of overall morbidity. We also evaluated the percentage of each complication after hospital discharge (Table 3). Among the complications, anastomosis stricture showed the highest proportion of PD incidence, with 83.3% of all anastomosis stricture complications occurring after hospital discharge. Other complications with >25% PD incidence included gastric stasis (25.0%), wound complication (27.6%), intra-abdominal abscess (31.1%), and stomach necrosis (50.0%).

**TABLE 3:**
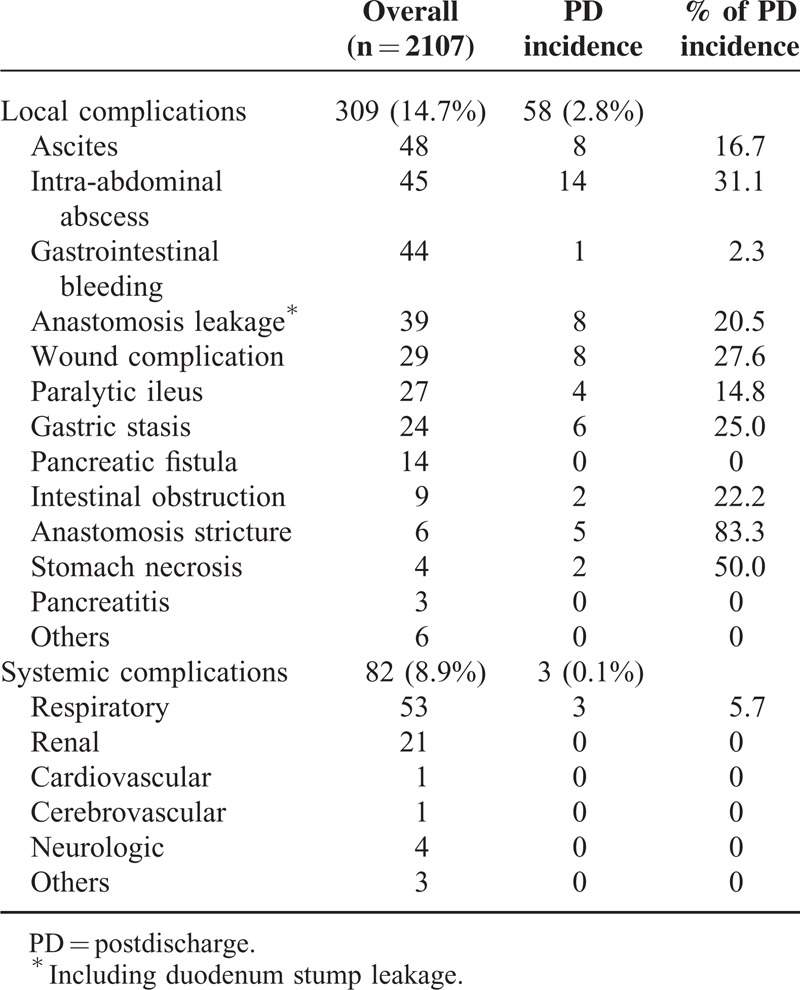
Complication Details

Table 4 summarizes the types and severities of 30-day PD complications. The most common PD complication was intra-abdominal abscess (n = 14), followed by wound complication, ascites, and anastomosis leakage (n = 8 each). The mean time to PD complications was 9.5 ± 4.7 days after hospital discharge. Fifty patients (2.4%) were readmitted to the hospital, and 7 (0.3%) underwent reoperation, which included patients who experienced anastomosis leakage (n = 3), stomach necrosis (n = 2), intra-abdominal abscess (n = 1), and intestinal obstruction (n = 1). No mortality occurred from PD complications.

**TABLE 4:**
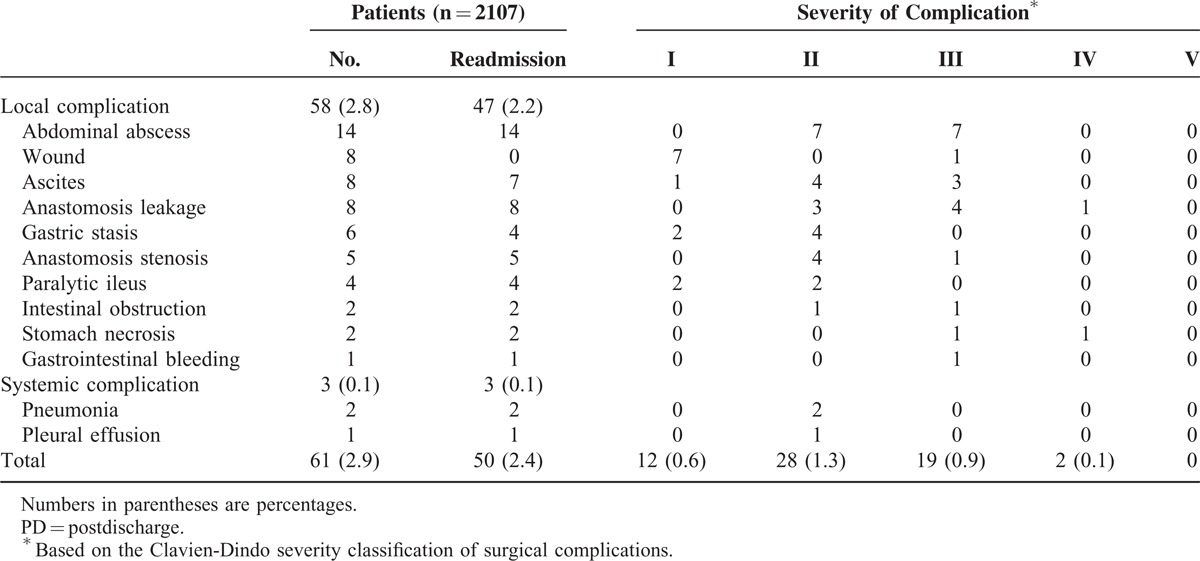
Types and Severities of 30-day PD Morbidity

In the subgroup of patients with total gastrectomy (n = 418), the incidence of 30-day PD complications was 2.6% (n = 11), and among them 10 patients (2.4%) were readmitted. The most common complication was intra-abdominal abscess (n = 4), followed by anastomosis stenosis (n = 2). One patient with intra-abdominal abscess and one patient with anastomosis stenosis underwent radiologic intervention (pig-tail insertion) and endoscopic intervention, respectively. Others were successfully managed with conservative care only (Table 5).

**TABLE 5:**
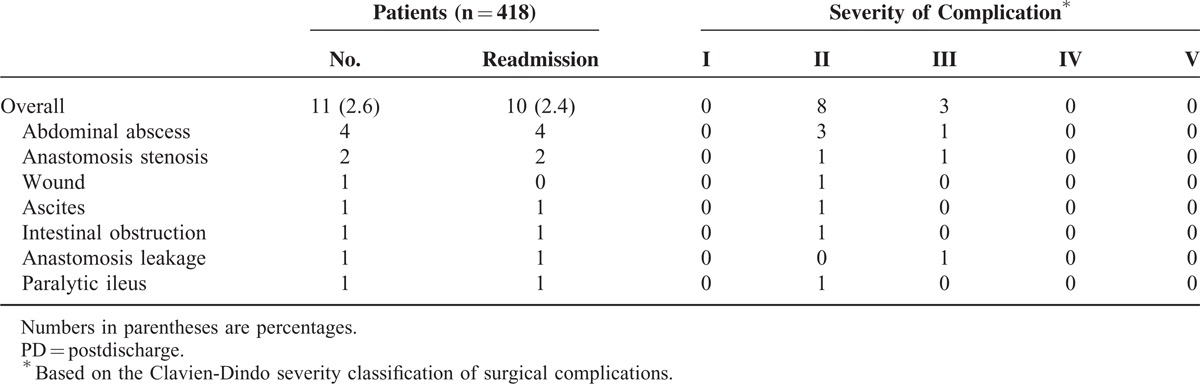
30-Day PD Morbidity After Total Gastrectomy

### Predictive Factors for 30-Day Postdischarge Morbidity

To identify predictive factors for 30-day PD morbidity, univariate and multivariate analyses were performed using each patient's demographic features, operative factors, and pathologic stages (Table 6). In the univariate analysis, the number of comorbidities and BMI were significantly associated with development of PD complications, while age, gender, operative techniques, operating time, hospital stay, and tumor stage were not. In the multivariate analysis adjusting all the variables, the number of comorbidities and BMI remained independent predictive factors for 30-day PD morbidity.

**TABLE 6:**
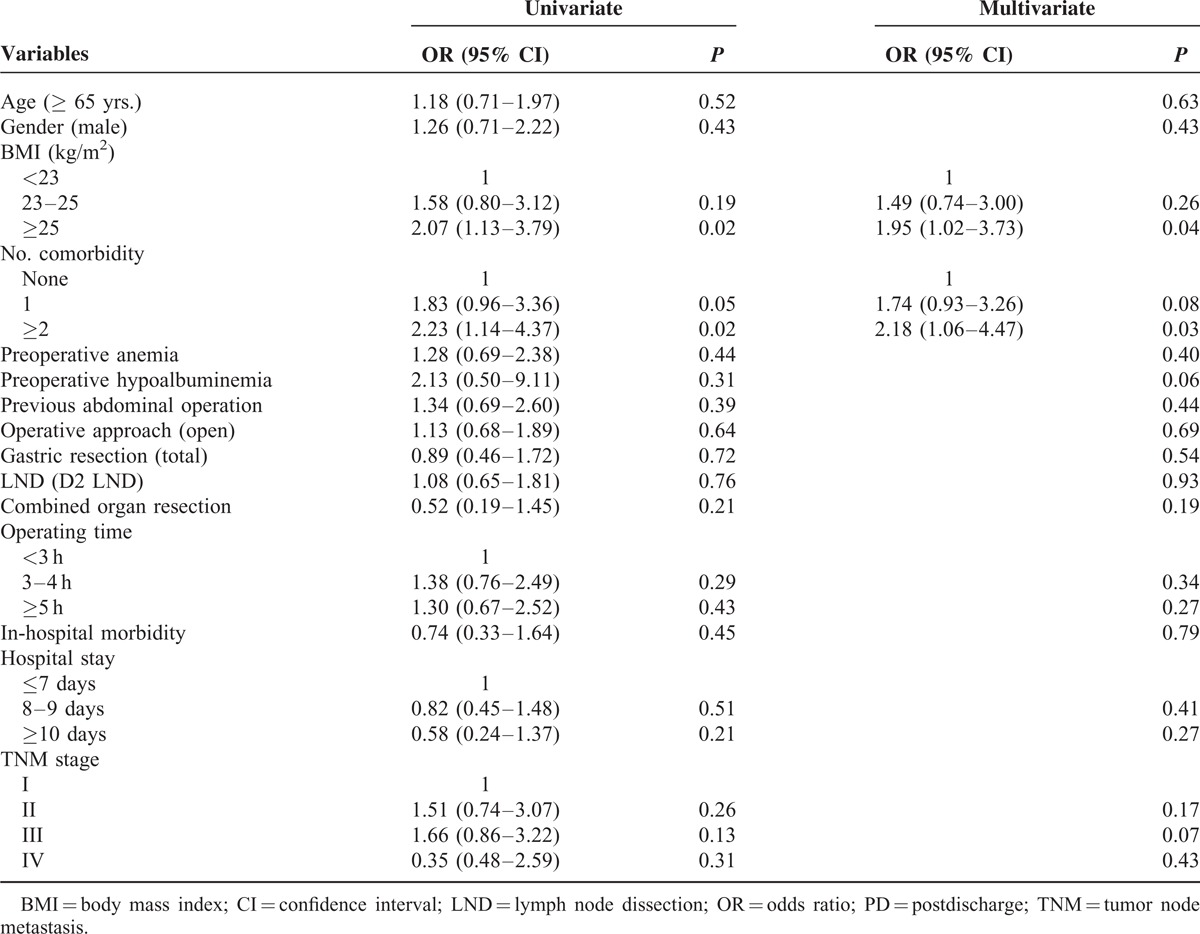
. Results of Univariate and Multivariate Analyses of Predicting Factors of 30-day PD Morbidity

Because the presence of comorbidity was found to be an independent predictive factor for 30-day PD morbidity, we further investigated the impact of each type of comorbidity on developing PD morbidity (Table 7). In the univariate and multivariate analyses, hypertension (odds ratio [OR] = 1.90, 95% confidence interval [CI] = 1.12–3.22) and liver cirrhosis (OR = 3.13, 95% CI = 1.15–8.48) were found to be significantly associated with PD complications, while other comorbidities were not.

**TABLE 7:**
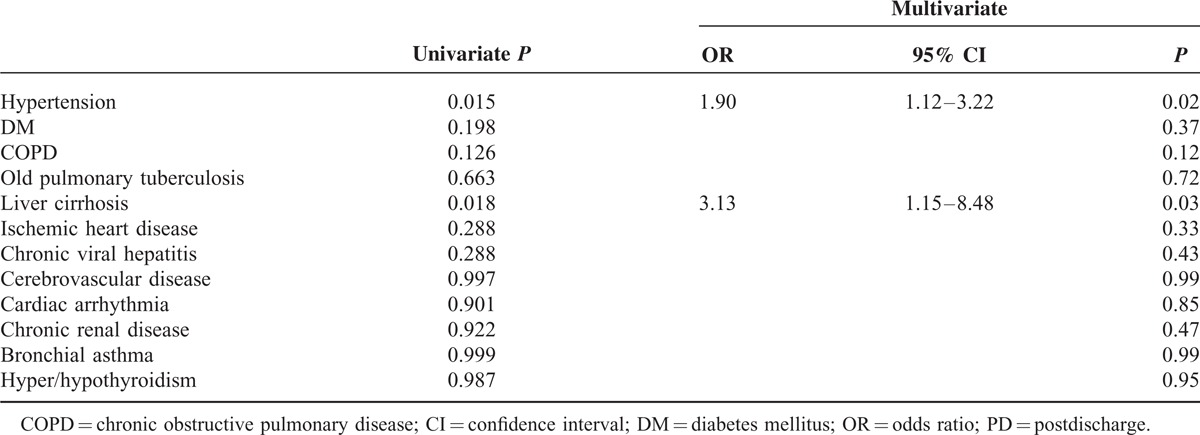
Impact of Each Type of Comorbidity on 30-day PD Morbidity

## DISCUSSION

PD complications and readmission significantly affect postoperative recovery and quality of life in surgical patients as well as increase healthcare costs. Accordingly, hospital readmission is increasingly used as a healthcare service indicator while monitoring the quality of patient care.^23,24^ Appropriate quality improvement initiatives focused on this issue necessitate comprehensive information about PD events. However, very limited data are available regarding how frequently or which types of complications occur after hospital discharge in patients undergoing abdominal surgical procedures. To the best of our knowledge, this is the first study investigating the incidence, types, and risk factors of PD complications after gastric cancer surgery. The present study prospectively collected data concerning PD complication in a large patient cohort and showed that a substantial proportion of complications (16.6% of postoperative morbidity) occurred during the early PD period. Therefore, an appropriate discharge plan including easy access to medical care for any PD complications is required to improve the quality of surgical care.

PD morbidity and readmission are often presented as a measure of hospital performance, but it may also be an important indicator of the performance of the healthcare system. In the United States, almost one-fifth (19.6%) of the Medicare beneficiaries discharged from a hospital were rehospitalized within 30 days, including 16.6% of patients undergoing major bowel surgery.^1^ Medicare payments for unplanned rehospitalization in 2004 were estimated at approximately $17.4 billion among the $102.6 billion of total hospital payments from Medicare.^25^ Given its attendant clinical and economic burdens to the healthcare system, the Medicare Payment Advisory Commission has suggested complementary changes in payment rates so that hospitals with high risk-adjusted readmission rates receive a lower average payment per case.^3^ In South Korea, healthcare is provided by a compulsory National Health Insurance system, and efforts are being made to improve the efficiency of the healthcare system at the governmental level. More recently, information about hospital quality, such as surgical morbidity and mortality, is being reported publicly, so that patients and referring providers can select hospitals based on performance rankings from these quality assessments. However, hospital readmission data are not provided in the quality assessment, and no comprehensive analysis of the socioeconomic burden of readmission on the healthcare system is available. Considering the significant effects of PD morbidity and mortality on hospital surgical quality,^26^ inclusion of PD events should be mandatory for proper quality assessment and correct information for the public.

Morbidity and mortality are major indicators of surgical performance. Although postoperative morbidity and mortality are commonly defined as any events occurring as an inpatient or as an outpatient within 30 days of the index operation, a structured review examining postoperative outcomes found that only 22% of reports included outpatient events.^27^ Some may argue that time-intensive collection of PD morbidity and mortality data is not necessary because collection of PD data can be particularly laborious and costly compared with collection of inpatient data. However, studies clearly showed that inclusion of PD events is important in the evaluation of surgical outcomes. Kazaure et al^2^ showed that nearly 40% of complications occurred after hospital discharge in general surgical procedures. Encinosa et al^28^ found that the morbidity after gastric bypass surgery increased by 81% when outpatient events were included. In addition, inclusion of PD complications significantly affects the measurement of the quality of patient care. In the study by Bilimoria et al^26^, including PD morbidity and mortality results in substantially changed hospital performance rankings compared with including in-hospital outcomes alone.

PD morbidity and readmission after gastrectomy were rarely examined in the previous studies. Kim et al^17^ investigated 5-year readmission after distal gastrectomy and reported 7.5% of patients were readmitted within 30 days after hospital discharge, with delayed gastric emptying and wound infection being the most common causes. Similarly, in the analysis of ACS-NSQIP data files, the 30-day PD complication rate was reported as 8.3% after gastrectomy, which constituted 29.9% of overall morbidity.^26^ In their study, surgical site infection accounted for nearly 50% of PD complications after gastrectomy. Other small studies reported 30-day readmission rates ranging from 11% to 14.6% after gastrectomy.^15,16^ Our study showed relatively lower PD morbidity (2.9%) and readmission rates (2.4%) compared with the previous reports. Several factors may explain this difference. The underestimation of PD events is less likely a possible explanation for this considering the prospectively collected data in the present study. Instead, this may be explained by the fact that this study is based on the data of a single large center that is specialized for gastric cancer surgery. Numerous studies have demonstrated considerable hospital-level variability in postoperative surgical outcomes according to the volume of surgery and specialization.^29,30^ In addition, it may be attributable to our longstanding practice of strict application of discharge criteria to the hospital discharge decision. Several studies have shown that accurate assessment of postoperative recovery status and the appropriate hospital discharge decision is essential to reduce PD morbidity and readmission.^31^ Collectively, based on our results and previous studies, PD complication rates may vary widely ranging from 3% to 15% after gastric cancer surgery, depending on the differences in patient population, surgical volume, or hospital setting.

The ideal way to minimize adverse PD events would be to adopt tailored discharge plans according to the risk of PD complications. If patients at risk for PD complications can be identified before discharge, early intervention or prolonged observation may decrease readmission risk and potential morbidity.^31,32^ The present study sought to determine the predictive factors for PD morbidity and found that risk factors for PD complications tended to be patient-related rather than disease- or operation-related. The presence of comorbidity (especially hypertension and liver cirrhosis) and obesity was significantly associated with PD morbidity in the univariate and multivariate analyses, whereas operative techniques, such as type of resection and LND, operative approach (open vs. laparoscopy), or operating time did not significantly influence the incidence of PD complications. Similar to our results, Ahmad et al^15^ reported preexisting cardiopulmonary disease was one of the main factors associated with higher 30-day readmission after gastrectomy. Obesity also has been reported to increase PD morbidity after general surgical procedures, along with diabetes mellitus and steroid use.^2^ However, other factors, such as open surgery, total gastrectomy, longer postoperative stay, and inpatient complication, showed results that are inconsistent with our study.^15,17^ We believe that large multi-institutional data with consistent surgical disciplines are required to develop appropriate prediction models with high reliability and validity.

Although this study investigated PD complications and readmission, other PD events, for example, nutritional disorder or functional gastrointestinal problems, were not captured in the database. Appropriate patient support for those functional problems is also essential for improving the quality of surgical care. Second, this study is also limited because the data are from a single tertiary referral center with a specialized gastric cancer division. Therefore, the PD morbidity and readmission rates observed in this study are less likely to be reproduced in hospitals with small volumes of gastric cancer surgery. PD morbidity and readmission rates may vary widely according to the volume of surgery and surgical practices. However, the present study still emphasizes the need for appropriate care plans for PD events, showing substantial proportion of PD morbidity regardless of surgical experience.

In conclusion, this study investigated complications and readmission after hospital discharge in a large group of patients undergoing gastric cancer surgery. We demonstrated that a substantial proportion of complications occurred in the early PD period, and patient-related factors, such as comorbidity and obesity, are associated with an increased risk of PD complications. Appropriate discharge plans based on identifying patients at increased risk as well as providing easy access to medical care for any complications are required to improve the quality of surgical care after gastric cancer surgery.
